# Discovery of Novel AKT Inhibitors with Enhanced Anti-Tumor Effects in Combination with the MEK Inhibitor

**DOI:** 10.1371/journal.pone.0100880

**Published:** 2014-06-30

**Authors:** Melissa Dumble, Ming-Chih Crouthamel, Shu-Yun Zhang, Michael Schaber, Dana Levy, Kimberly Robell, Qi Liu, David J. Figueroa, Elisabeth A. Minthorn, Mark A. Seefeld, Meagan B. Rouse, Sridhar K. Rabindran, Dirk A. Heerding, Rakesh Kumar

**Affiliations:** 1 Oncology R & D, GlaxoSmithKline, Collegeville, Pennsylvania, United States of America; 2 Platform Technology & Science, GlaxoSmithKline, Collegeville, Pennsylvania, United States of America; Florida International University, United States of America

## Abstract

Tumor cells upregulate many cell signaling pathways, with AKT being one of the key kinases to be activated in a variety of malignancies. GSK2110183 and GSK2141795 are orally bioavailable, potent inhibitors of the AKT kinases that have progressed to human clinical studies. Both compounds are selective, ATP-competitive inhibitors of AKT 1, 2 and 3. Cells treated with either compound show decreased phosphorylation of several substrates downstream of AKT. Both compounds have desirable pharmaceutical properties and daily oral dosing results in a sustained inhibition of AKT activity as well as inhibition of tumor growth in several mouse tumor models of various histologic origins. Improved kinase selectivity was associated with reduced effects on glucose homeostasis as compared to previously reported ATP-competitive AKT kinase inhibitors. In a diverse cell line proliferation screen, AKT inhibitors showed increased potency in cell lines with an activated AKT pathway (via *PI3K/PTEN* mutation or loss) while cell lines with activating mutations in the MAPK pathway (*KRAS/BRAF*) were less sensitive to AKT inhibition. Further investigation in mouse models of KRAS driven pancreatic cancer confirmed that combining the AKT inhibitor, GSK2141795 with a MEK inhibitor (GSK2110212; trametinib) resulted in an enhanced anti-tumor effect accompanied with greater reduction in phospho-S6 levels. Taken together these results support clinical evaluation of the AKT inhibitors in cancer, especially in combination with MEK inhibitor.

## Introduction

Activation of the PI3K-AKT pathway is common in many human malignancies leading to an increase in cell survival, growth and proliferation; all necessary hallmarks of a cancer cell. This pathway is up-regulated in cancer cells as a result of a variety of genetic alterations including over-expression of or activating mutations in receptor tyrosine kinases (e.g. *ERBB2* or *MET*), activating mutations in PI3K subunits, loss or promoter methylation of *PTEN* and over-expression of or mutations in the *AKT*s [1]. The AKTs are a family of serine-threonine kinases and an integral component in the signaling cascade downstream of PI3K and PTEN which work to catalyze the formation of PIP3 membrane lipids from PIP2 and back, respectively. PIP3 lipids tether the AKT kinases to the membrane via their plextrin homology domain which enables activation by phosphorylation on Thr308 by PDK1 and Ser473 by the mTORC2 complex [2]–[7]. Activated AKT phosphorylates a variety of proteins (e.g. FOXO, TSC1/2, PRAS40, GSK3β) involved in cell survival, growth and proliferation. Given the importance of this pathway in various cancers, a number of small molecules targeting PI3K with and without mTOR inhibition are being evaluated in patients [8]. Several AKT inhibitors have also entered clinical development; however some of them block activation of AKT rather than inhibiting kinase activity [9]–[13].

We report a novel class of orally available, ATP-competitive, pan-AKT kinase inhibitors, GSK2110183 and GSK2141795. Both molecules inhibit the AKT pathway and proliferation of various tumor cells in vitro and in vivo. In addition, these compounds have more favorable glucose homeostasis profiles than some previously reported AKT inhibitors [9]–[12]. Consistent with AKT pathway inhibition in pre-clinical mouse models of cancer, treatment of tumor bearing mice with GSK2110183 or GSK2141795 resulted in tumor growth inhibition and/or regression. Combination of AKT inhibitor with a MEK inhibitor, GSK1120212, resulted in increased efficacy compared to either single agent independently. These data support the development of these AKT inhibitors for clinical use alone and in combination with a MEK inhibitor.

## Materials and Methods

### Drugs and Materials

GSK2110183 and GSK2141795 were synthesized at GlaxoSmithKline (Figure S1). Compounds were dissolved in DMSO at 10 mmol/L prior to use for all *in vitro* studies. For *in vivo* use, both compounds were formulated in 20% polyethylene glycol (PEG) 400/1% DMSO. GSK690693 [12], was used as a reference molecule in vivo and formulated in 5% dextrose. GSK1120212 (trametinib), a selective MEK inhibitor [14], was formulated in 0.5% hydroxypropylmethylcellulose (Sigma) and 0.2% Tween-80 pH 8.0.

### Animals

Eight to twelve week old female nu/nu CD-1 mice (Charles River Laboratories) and severe combined immunodeficient (SCID) mice (Taconic Farms) were used. All animal studies were conducted after review by the Institutional Animal Care and Use Committee at GSK and in accordance with the GSK Policy on the Care, Welfare and Treatment of Laboratory Animals. The Institutional Animal Care and Use Committee at GSK specifically approved these studies.

### Cell lines and culture

Cell lines were purchased from the American Type Culture Collection (Manassas, VA) or from DSMZ, the German Resource Center for Biological Material (Braunschweig, Germany). Cells were routinely cultured in the recommended growth medium containing 10% FBS. All cell lines were maintained in humidified incubators at 37°C under 5% CO2. The cell lines used in this study were not authenticated by any tests in our laboratory.

### Kinase Assays

The potency of compounds against AKT enzymes was measured as described before [12]. Since GSK2110183 and GSK2141795 are highly potent inhibitors of the 3 isoforms of AKT, the true potency (K_i_^*^) of the inhibitors was initially determined at low enzyme concentrations (0.1 nM AKT1, 0.7 nM AKT2, and 0.2 nM AKT3) using a filter binding assay and then confirmed with progress curve analysis. In the filter binding assay, a pre-mix of enzyme plus inhibitor was incubated for 1 h and then added to a GSKα peptide (Ac-KKGGRARTSSFAEPG-amide) and [γ^33^P] ATP. Reactions were terminated after 2 h and the radio labeled AKT peptide product was captured in a phospho-cellulose filter plate. Progress curve analysis utilized continuous real-time fluorescence detection of product formation using the Sox-AKT-tide substrate (Ac-ARKRERAYSF-d-Pro-Sox-Gly-NH2).

GSK2110183 and GSK2141795 were tested against a diverse panel of kinase assays at GlaxoSmithKline and Millipore. Initially, the compounds were tested at 0.5 and 10 µM in all available kinase assays and were followed up with full IC_50_ curves against a subset of enzymes that showed strong inhibition against 0.5 µM, for which in-house assay were not available.

### ELISA Assay

A phospho-GSK3β ELISA was used to determine the EC_50_ for GSK2110183 and GSK2141795 in human cell lines. ELISA plates were prepared by coating with anti-GSK3β antibody (R&D Systems), and blocked with 5% Milk/0.1% Tween-20. Cells were seeded at 25,000 cells/96-well overnight and treated with DMSO or various concentrations of drug for 1 h. Cells were lysed in 20 mM Tris-HCl (pH 8.0), 137 mM NaCl, 2 mM EDTA, 10% glycerol and 1% Triton X-100 and lysates transferred to ELISA plates and incubated overnight. The plates were washed and incubated with rabbit anti-phospho-GSK3β (Ser9) antibody (R&D Systems) for 1 h. After washing, plates were developed using HRP-linked anti-rabbit IgG, and 3,3′,5,5′-tetramethylbenzidine as substrate. Absorption was measured in a microplate spectrophotometer at 450 nm. The phospho-PRAS40 ELISA followed similar methodology as above with the exception of using a commercially available PRAS40 (pThr246) kit (Biosource) and following manufacturers' instructions for the ELISA analysis.

### Proliferation Assays

Various tumor cell lines were obtained from different sources including; the American Type Cell Culture Collection, the Developmental Therapeutics Program, the National Cancer Institute, the German Resource Center for Biological Material and the European Collection of Animal Cell Cultures. Cell lines were typically grown in RPMI 160 medium containing 10% FBS. Some cell lines were grown in media specified by the vendor. A 3-day proliferation assay using CellTiter-Glo (CTG, Promega) was performed to measure the growth inhibition by the compounds at 0–30 µM. Cell growth was determined relative to untreated (DMSO) controls. EC_50_'s were calculated from inhibition curves using a 4- or 6-parameter fitting algorithm in the Assay Client application.

### Western Analysis

Cells treated with DMSO or compounds for different duration were lysed using RIPA buffer (Teknova) containing protease and phosphatase inhibitors (Sigma Aldrich). Equal amounts of protein were resolved in 4–12% SDS-PAGE gels (Invitrogen), transferred onto 0.45 µm PVDF membrane (Invitrogen), and incubated with primary antibodies (1∶1000 dilution) overnight. Antibodies for AKT, phospho-AKT (Ser473 and Thr308), phospho-FOXO (Thr24/32), phospho-MEK1/2 (Ser217/221) were purchased from Cell Signaling Technologies. Phospho-GSK3β (Ser9) antibody was from R&D Systems. PRAS40 and phospho-PRAS40 (Thr246) antibodies were obtained from Millipore. Phospho-ERK1/2 (Tyr204), ERK1/2, and phospho-Caspase 9 (Ser196) antibodies were purchased from Santa Cruz Biotechnologies. Tubulin antibodies were purchased from Sigma Aldrich. Following incubation with primary antibody, blots were washed and incubated with IRDye-680 goat-anti-mouse or IRDye-800CW goat-anti-rabbit antibodies (1; 10,000 dilution; LI-COR) for 1 h. Following thorough washing, blots were analyzed using an infrared imaging system (LI-COR).

### *In vivo* Xenograft experiments

Tumors were initiated by injecting either cells (SKOV3, CAPAN-2 and HPAC) or a tumor fragments (BT474) subcutaneously into 6–8 week female athymic nude (SKOV3) and SCID (all others) mice. Once tumors reached between 120 and 300 mm^3^, mice were randomized according to tumor volume into groups of n = 7–10 mice per treatment. GSK2110183 and GSK2141795 were administered daily at various doses by oral gavage. In combination experiments, GSK1120212 was also administered daily by oral gavage. Tumor volumes and body weight were measured twice weekly, tumor volume was measured with calipers and calculated using equation: Tumor volume (mm^3^)  =  (length x width)^2^/2. Results are represented as percent inhibition on completion of dosing  = 100 x [1- average growth of drug-treated population/average growth of vehicle-treated control population].

### *In vivo* dose response pharmacodynamic assay

SCID mice bearing BT474 tumor xenografts were treated with either vehicle, GSK2110183 or GSK2141795 daily for 7 days prior to harvesting tissue 2 h post the last dose. Protein lysates were analyzed by phospho-PRAS40 ELISA according to the methods described above. Concentration of the test compounds in the tissue and blood was analyzed using protein precipitation with acetonitrile, followed by HPLC/MS/MS analysis using positive ion atmospheric pressure chemical ionization or Turbo ionspray ionization (API 4000 or API 5000, Applied Biosystems). The lower level of detection of compound was 10 ng/mL and the assays were linear over a 100- to a 1000-fold drug concentration range.

Blood glucose levels were determined from tail vein puncture in mice using a handheld glucometer. Plasma Insulin levels were using the Rat Ultrasensitive Insulin ELISA kit (Crystal Chem Inc).

### Immunohistochemistry

Mice bearing HPAC tumor xenografts were treated with 30 mg/kg GSK2141795 and 0.3 mg/kg of GSK1120212 daily for 3 days (n = 3/group). Four hours following the third dose, tissues were harvested and fixed in 10% neutral buffered formalin for 24 h, and then paraffin embedded. Sections were cut (6 µM) and immunohistochemical analysis was carried using the Discovery XT system (Ventana). Briefly, sections were deparaffinized, hydrated and loaded on the Discovery XT. Antigen retrieval was performed using Tris based (EDTA) buffer solution, CCL (Ventana) at 95–100°C for 20–40 min. Endogenous peroxidases were quenched using the Inhibitor-D 3% H_2_O_2_ reagent (Ventana) for 4 min at 37°C. Primary antibodies included Ki67 (30–9; Ventana), phospho-ERK (MAPK-YT; Sigma) and Thr202/Tyr204 phospho-ERK (D13.14.4E), CC3 (Asp175), PRAS40 (D23C7), Thr-246 phospho-PRAS40 (C77D7), ERK (137F5), AKT (C67E7), Ser-473 phospho-AKT (D9E), S6 (54D2), Ser235/236 phospho-S6 (D57.2.2E) all purchased from Cell Signaling. Diluted antibodies were applied to the sections for 8 h then detected using OmniMap HRP anti-Rb or OmniMap HPR anti-mouse reagent and the ChromaMap DAB detection kit (Ventana). Tissues were counterstained with Harris' hematoxylin (Lerner Laboratories), dehydrated, cleared, and coverslipped. Images were acquired with an Axio Imager D2 microscope (Zeiss) equipped with an Axiocam HRc digital camera (Zeiss). Image analysis was carried out using the MetaMorph Imaging program (Molecular Devices).

### Statistical Analysis

Mann Whitney non-parametric t-test was used to analyze association of various genetic alterations in cell lines and their sensitivity to compounds in cell proliferation assay.

## Results

### GSK2141795 and GSK2110183 are potent and selective inhibitors of the AKT kinases which downregulate pathway activity resulting in cell cycle arrest and cell death

GSK2110183 and GSK2141795 are ATP competitive, time dependant and fully reversible inhibitors of the AKT kinase family. GSK2110183 has a Ki^*^ of 0.08, 2 and 2.6 nM against AKT1, AKT2 and AKT3, respectively, and GSK2141795 has a Ki^*^ of 0.066, 1.4 and 1.5 nM against AKT1, AKT2 and AKT3, respectively (Table 1). Both compounds inhibit the kinase activity of the E17K AKT 1 mutant protein in a standard kinase assay with EC_50_'s of 0.2 nM. Kinase selectivity was evaluated at 0.5 and 10 µM compound concentration against a panel of 261 different kinase assays, including >225 unique kinases together with some mutant forms and some orthologs from mouse, rat or yeast origin. The majority of the enzymes tested (∼90%) showed <50% inhibition at 0.5 µM of both compounds (Table S1). Most of the enzymes that were inhibited >50% at 0.5 µM belonged to the AGC family including PKA, PKC, and PKG isoforms. IC_50_ values were generated for these enzymes (Table 1).

**Table 1:** Biochemical and Cellular Activity of GSK2110183 and GSK2141795.

Biochemical Assay		IC_50_ (nM)	
Kinase#		GSK2110183	GSK2141795
AKT1		0.08*	0.066*
AKT2		2*	1.4*
AKT3		2.6*	1.5*
AKT1 E17K mutant		0.2	0.2
P70S6K		251	50
PKA		1.3	2.0
PKCα		>1000	>1000
PKCβ1		430	56
PKCβ2		>1000	86
PKCδ		1000	69
PKCγ		>1000	200
PKCε		>1000	>1000
PKCθ		510	64
PKCη		210	49
PKCι		>1000	>1000
PKCμ		>1000	690
PKCζ		>1000	>1000
PKG1α		0.9	<1
PKG1β		4.0	<1
ROCK		100	126
RSK1		316	200
**Cellular Assay** $	**Cell Line**		
pGSK-3β	BT474	316±91	143±21
pGSK-3β	LNCaP	76±16	34±5
pPRAS40	BT474	121±40	39±0.5
pPRAS40	LNCaP	104±5	55±2
FOXO_GFP translocation	MDA-MB-468	52±11	47±37

# IC_50_ or K_i_ of selected kinases inhibited >50% at 0.5 µM from larger kinase panel.

*Data represents K_i_ (nM) values.

$ Data represents mean ± std dev from multiple experiments for cellular assays.

**Figure 1:**
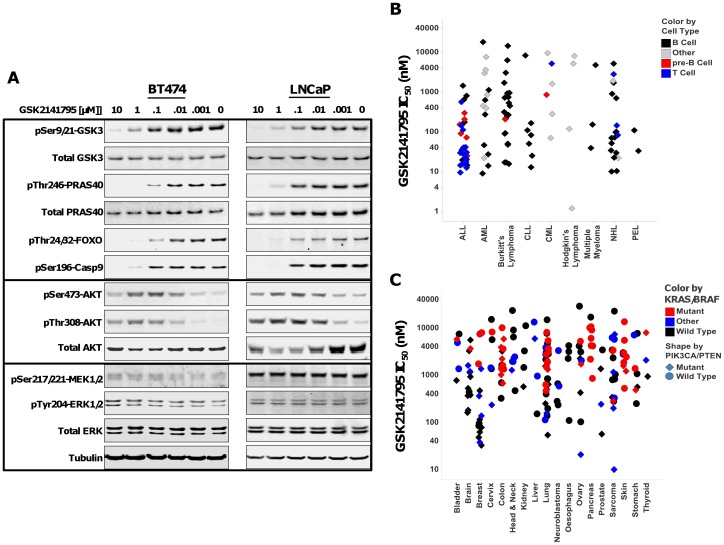
Effect of GSK2141795 on AKT signaling and growth inhibition in human cancer cell lines. *A*, BT474 (left) and LNCaP (right) cell lines were treated with DMSO or GSK2141795 for 1 h. Western analysis was performed to assay levels of phosphorylated and total GSK-3, PRAS40, AKT and ERK, phosphorylated FOXO, Caspase 9, and MEK. Tubulin was used as a loading control. *B*, Scatter plot of EC_50_'s for anti-proliferative effect of GSK2141795 against various haematological cancer cell lines. Cell lines are grouped according to their disease classification and sub-divided into cellular origin of B cell (black diamond), pre-B cell (red diamond), T cell (blue diamond) or other (grey diamond). *C*, Scatter plot of EC_50_'s for various solid cancer cell lines treated with GSK2141795. Cells were treated as described above. Cell lines are grouped by tissue of origin then further divided by genetic status. *KRAS/BRAF^V600E^* mutation status is represented by color with mutant (red), wild type (black) or other (blue); whereas *PIK3CA* or *PTEN* status represented with shape, mutant (diamond) and wild type (circle).

**Figure 2:**
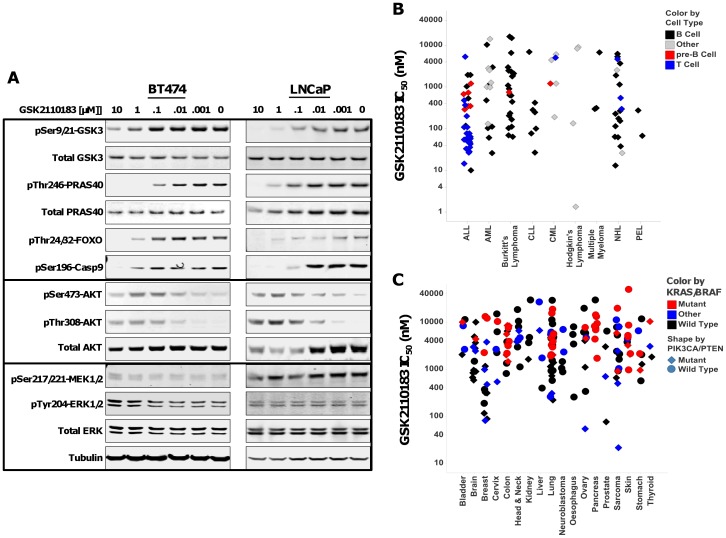
Effect of GSK2110183 on AKT signaling and growth inhibition in human cancer cell lines. *A*, BT474 (left) and LNCaP (right) cell lines were treated with DMSO or GSK2110183 for 1 h. Western analysis was performed to assay levels of phosphorylated and total GSK-3, PRAS40, AKT and ERK, phosphorylated Foxo, Caspase 9, and MEK. Tubulin was used as a loading control. *B*, Scatter plot of EC_50_'s for anti-proliferative effect of GSK2110183 against various haematological cancer cell lines. Cell lines are grouped according to their disease classification and sub-divided into cellular origin of B cell (black diamond), pre-B cell (red diamond), T cell (blue diamond) or other (grey diamond). *C*, Scatter plot of EC_50_'s for various solid cancer cell lines treated with GSK2110183. Cells were treated as described above. Cell lines are grouped by tissue of origin then further divided by genetic status. *KRAS/BRAF^V600E^* mutation status is represented by color with mutant (red), wild type (black) or other (blue); whereas *PIK3CA* or *PTEN* status represented with shape, mutant (diamond) and wild type (circle).

Whole cell assays were performed to test the ability of GSK2110183 and GSK2141795 to inhibit the kinase activity of AKT in human tumor cell lines. Two cell lines, BT474 (breast; *ERBB2*+, *PIK3CA* K111N) and LNCaP (prostate; *PTEN* null), were chosen for this analysis due to their AKT pathway activation. GSK2141795 and GSK2110183 showed concentration-dependent effect on multiple AKT substrate phosphorylation levels, including GSK3β, PRAS40, FOXO and Caspase 9 in both cell lines (Figure 1A, Figure 2A, Table 1). As reported with other ATP-competitive AKT kinase inhibitors [12], [15], both compounds show a concentration-dependent feedback increase in AKT phosphorylation. There was no change in phosphorylation of MEK and ERK1/2 in these cell lines at 1 h suggesting selective inhibition of AKT pathway (Figure 1A, Figure 2A).

Phosphorylation of the transcription factor FOXO3 by AKT regulates its cellular localization, and inhibition of AKT leads to unphosphorylated FOXO3A that translocates to the nucleus [16]. A GFP tagged FOXO3A allows the visualization and quantification of this translocation in the presence of varying concentrations of an AKT inhibitor (Methods S1). The EC_50_ values for GSK2110183 and GSK2141795 in this assay were very similar at 53 and 47 nM, respectively (Table 1 and Figure S2).

### GSK2110183 and GSK2141795 preferentially inhibit the proliferation of human cancer cells lines with AKT pathway activation

The ability of AKT inhibitors to inhibit the growth of human tumor cell lines was assessed in a panel of 290 cells lines. Out of these, 112 of the cell lines were derived from various hematological malignancies while 178 were derived from solid tumors of different origins. Proliferation assays were carried out using a titration of compounds for three days and analyzed using the CTG assay. Individual EC_50_'s as well as relevant genetic mutations for all solid and hematological cell lines are listed in tables S2 and S3, respectively.

GSK2141795 and GSK2110183 consistently have more potent anti-proliferative effects on a variety of hematological malignancies. Overall 81% of the hematological cell lines were sensitive to GSK2141795 while 65% were sensitive to GSK2110183 (EC_50_<1 µM). A large proportion of B and T cell origin ALL, Non-Hodgkins Lymphoma (NHL) and CLL cell lines were particularly sensitive to AKT inhibition (Figure 1B, Figure 2B). Among solid tumor cell lines, 36% and 21% had EC_50_<1 µM in response to GSK2141795 and GSK2110183, respectively. Overall, the data shows that cell lines from breast and lung cancer are the most sensitive with 66–73% of the breast cell lines having EC_50_<1 µM to the AKT inhibitors and 20–34% of lung cell lines having EC_50_ values <1 µM (Figure 1C, Figure 2C). Interestingly, it appears that many of the cell lines sensitive to GSK2141795 and GSK2110183 have PI3K or PTEN alterations (EC_50_<1 µM; p<0.0001 for both compounds). Conversely, many of the more resistant cell lines (EC_50_>1 µM) contain activating mutations to *KRAS*, *NRAS* or *BRAF*, a finding that is statistically significant for GSK2141795 (p = 0.026) and GSK2110183 (p = 0.030; Figure 1C and Figure 2C).

**Figure 3:**
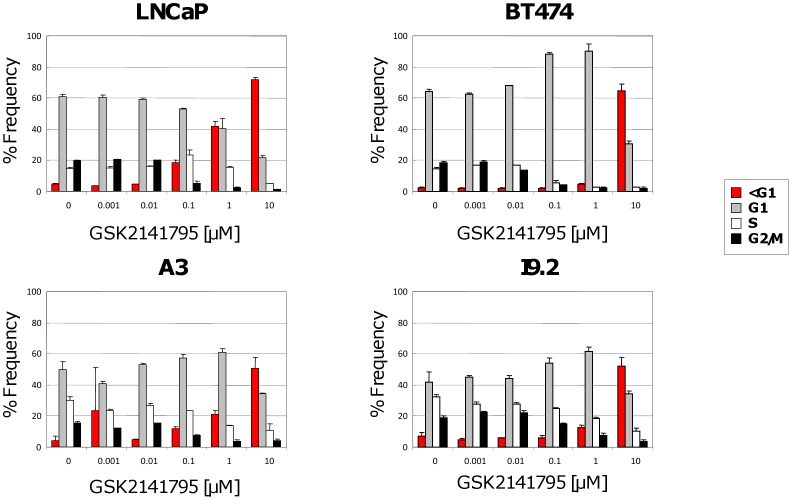
Effect of GSK2141795 on cell cycle. LNCaP, BT474, A3 and I9.2 cells lines were treated with GSK2141795 for 24; bars, SD.

To further characterize the cellular pharmacology, cell cycle analysis was performed with GSK2141795 using BT474, LNCaP, A3 and 19.2 cell lines. Treatment of BT474 cells resulted in G1 arrest which was evident at 1 µM, with an accumulation in <G1 observed at higher concentrations (10 µM) which is suggestive of cell death (Figure 3). LNCaP cells exposed to various concentrations of compound showed a concentration-dependent increase in cells within the sub-G1 phase which is indication of cell death with no obvious increase in G1 (Figure 3). The two hematological cell lines A3 and I9.2 showed a dose dependant increase in G1 cellular arrest at 1 µM similar to the BT474 cells (Figure 3). GSK2110183 had similar effects on the cell cycle to GSK2141795 (data not shown).

### The pharmacodynamic (PD) and pharmacokinetic (PK) relationship in mouse tumor models

**Figure 4:**
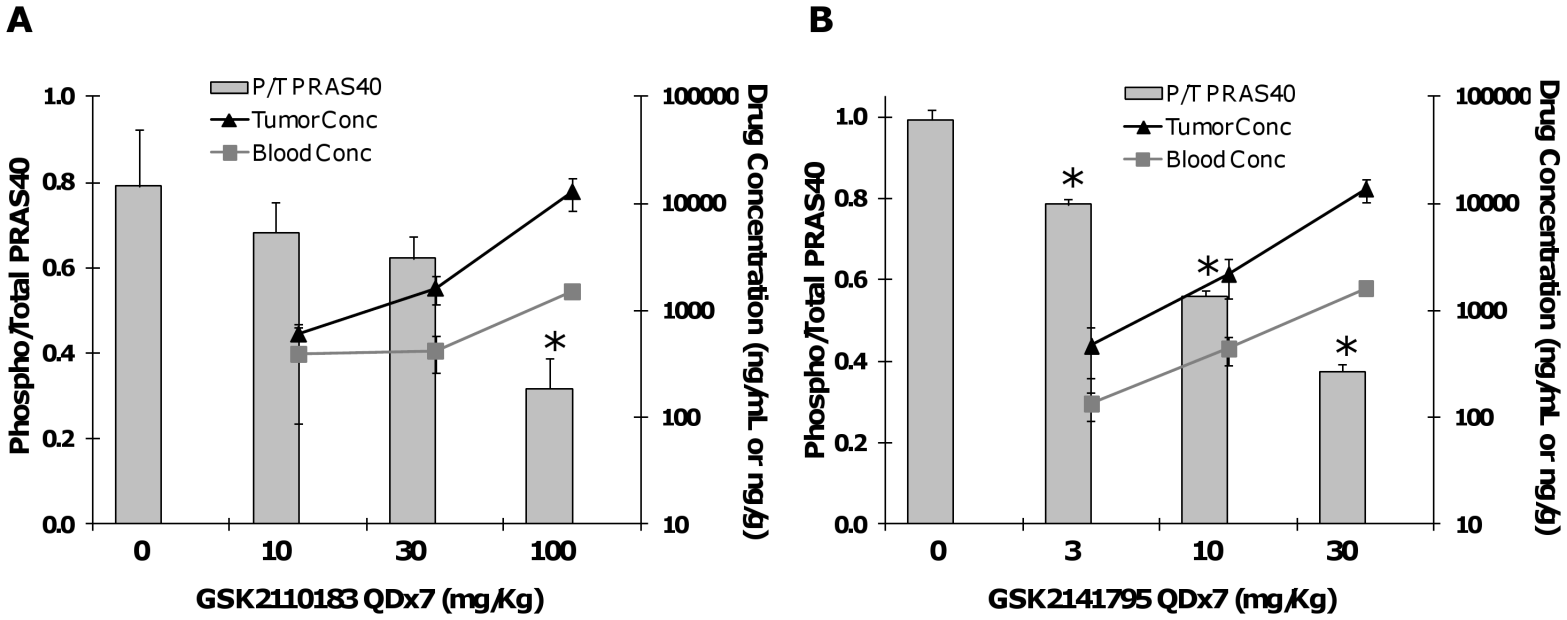
Dose responsive PD/PK relationship of GSK2110183 and GSK2141795 in BT474 tumor xenografts. Mice bearing BT474 tumors were treated with vehicle or GSK2110183 at 10, 30 or 100/kg (A) or GSK2141795 at 3, 10 and 30 mg/kg (B) daily for 7 days (QDx7; n = 3/group). Tumors were harvested and analysed by ELISA for phosphorylated and total PRAS40 levels. The concentration of compound in the tumor (black triangles; ng/g) and blood (grey squares; ng/mL) was quantified by LC/MS-MS. Data represents mean ± s.d. *p<0.01.

The ability of both orally available AKT kinase inhibitors to inhibit the phosphorylation of PRAS40 in tumors was assessed in mice bearing BT474 tumors. PRAS40 phosphorylation was decreased by 8%, 37% and 61% in tumors of mice dosed with GSK2110183 at 10, 30 and 100 mg/kg QDx7, respectively (Figure 4A). The concentration of GSK2110183 in the blood and tumor increased dose proportionally, with tumor exposure consistently higher than blood. Approximately 3 µM of circulating GSK2110183 (1500 ng/mL) corresponded with a 61% decrease in phospho-PRAS40. Similar experiments were performed for GSK2141795 and show a dose responsive inhibition of phospho-PRAS40 with a 30 mg/kg administration resulting in 62% inhibition corresponding to ∼3 µM of compound in the blood (Figure 4B).

A time course experiment with GSK2110183 at 100 mg/kg shows a sustained 60% inhibition of phospho-PRAS40 for 24 h with phospho-PRAS40 levels returning to baseline by 48 h (Figure S3A). Tumor GSK2110183 levels were higher than blood, and blood concentrations of 3–4 µM result in 60% inhibition of phospho-PRAS40. GSK2141795 show similar kinetics, maintaining 60% inhibition of phospho-PRAS40 for 8 h with levels returning to baseline by 48 h. Pharmacodynamic effects of GSK2141795 correlated with 2–3 µM blood concentrations maintained for 8 h (Figure S3B).

### Effects on glucose homeostasis

**Figure 5:**
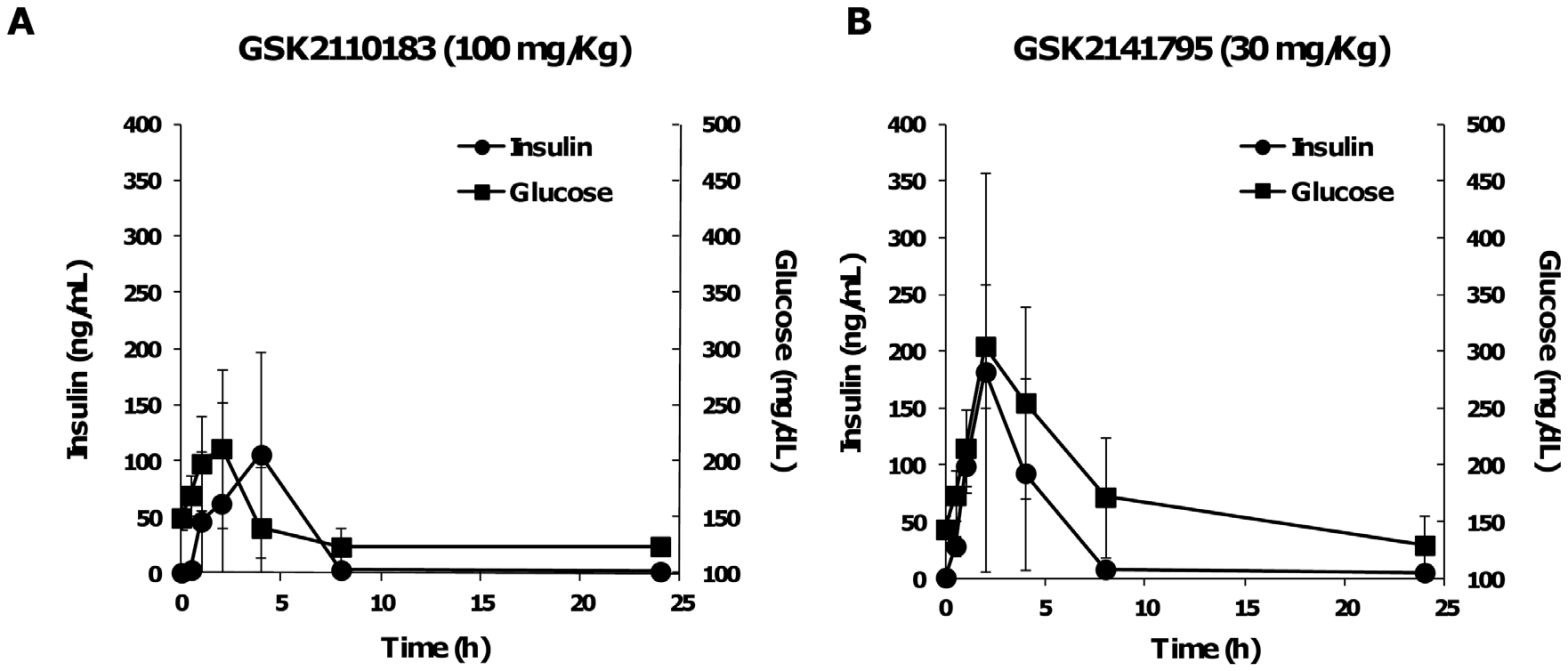
The impact of GSK2110183 and GSK2141795 on glucose homeostasis in vivo. Blood glucose (black squares) and plasma insulin (black circles) levels were assayed over time in mice treated with a single efficacious dose of GSK2110183 (30 mg/kg) or GSK2141795 (100 mg/kg). Data represents mean ± s.d.

The effect on blood glucose and plasma insulin was evaluated after a single oral dose, selected based on the respective maximally tolerated dose in a repeat dose study for 2–3 weeks in mice. Mice were administered 100 mg/kg GSK2110183 orally once and blood harvested 0, 0.5, 1, 2, 4, 8 and 24 h post dosing. Blood glucose levels were minimally affected with an average elevation to 211 mg/dL 2 h post dosing and a return to normal levels by 8 h (Figure 5A). Insulin levels increased to 105.6 ng/mL 4 h post dosing which returned to baseline by 8 h (Figure 5A). A single 30 mg/kg dose of GSK2141795 results in 200 mg/dL blood glucose levels 2 h post dosing and a concomitant elevation in plasma insulin (188 ng/mL; Figure 5B). Both glucose and insulin levels returned to normal by 8 h following administration of GSK2141795 (Figure 5B).

### The anti-tumor effect *in vivo*

**Figure 6:**
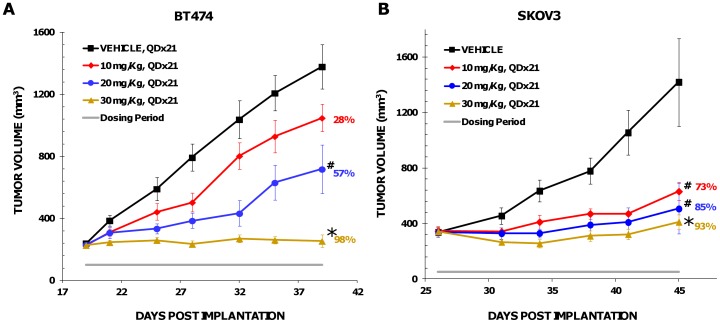
Anti-tumor activity of GSK2141795 in vivo. Mice bearing either BT474 (*A*) or SKOV3 (*B*) tumors were treated with vehicle (black line) or GSK2141795 at 10 (red line), 20 (blue line) or 30 (gold line) mg/kg, once daily for 21 d (QDx21). Duration of treatment is shown by the horizontal grey line. Tumor volume was measured twice per week. Data represents mean ± SEM. #p<0.05, *p<0.01.

Given the observed pharmacodynamic inhibition of the AKT pathway, the efficacy of both compounds was assessed in mice bearing established human tumor xenografts. Mice bearing BT474 tumors were dosed orally with either vehicle or GSK2141795. After 20 days of treatment with 10, 20 and 30 mg/kg of GSK2141795, tumor growth inhibition (TGI) of 28, 57 and 98%, respectively, was observed relative to vehicle control (Figure 6A). Minimal body weight loss of 3–8% was reported on day 6 of dosing which recovered by the end of study (data not shown). Similarly, mice bearing BT474 breast tumor xenografts were dosed orally with either vehicle or GSK2110183 at 10, 30 or 100 mg/kg daily for 21 days which resulted in 8, 37 and 61% TGI, respectively (Figure S4A). Mice tolerated GSK2110183 well, with 1–3% body weight loss reported after 5 days of dosing which recovered over the course of the study. Other tumor xenograft models which possess an activation of the AKT pathway were explored to further demonstrate compound efficacy. Mice bearing SKOV3 ovarian tumor xenografts with 10, 20 and 30 mg/kg GSK2141795 QD displayed a 73, 85 or 93% TGI, respectively, compared to vehicle control (Figure 6B). Similarly, mice treated with GSK2110183 at 10, 30 and 100 mg/kg resulted in 23, 37 and 97% TGI, respectively, of SKOV3 xenografts (Figure S4B).

### Combination with MEK inhibitor

**Figure 7:**
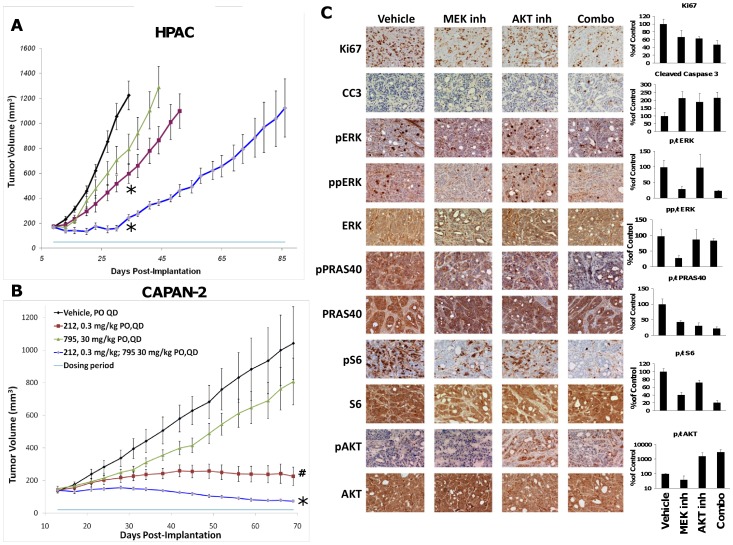
Combination anti-tumor effect of AKT and MEK inhibitors in mouse models of pancreatic cancer. Mice bearing HPAC (*A*) or CAPAN-2 (*B*) tumor xenografts were treated with either vehicle (black line), 0.3 mg/kg GSK1120212 (purple line), 30 mg/kg GSK2141795 (green line) or the combination of both (blue line). Data represents mean ± SEM. #p<0.05, *p<0.01. *C*, Immunohistochemical evaluation of HPAC tumor xenografts treated with vehicle, GSK1120212 at 0.3 mg/kg QDx3 (MEK inh), GSK2141795 at 30 mg/kg QDx3 (AKT inh) or the combination of GSK1120212 plus GSK2141795 at 0.3 and 30 mg/kg, respectively (combo). Tumor tissues were stained with markers of proliferation (Ki67), apoptosis (Cleaved Caspase 3; CC3), MAPK pathway activation (phospho-ERK, dually phospho-ERK and total ERK) and AKT pathway activation (phospho-PRAS40, total PRAS40, phospho-S6 kinase, total S6K, phospho-AKT and total AKT). Quantitative image analysis of various immune-staining is represented as a percentage of the vehicle control (100%). Data represents mean ± s.d.

GSK2141795 was tested in combination with the MEK inhibitor, GSK1120212, in pancreatic tumor xenografts to assess enhanced inhibition of tumor growth when the MAPK and AKT pathways are inhibited in unison [1], [17]. Mice bearing HPAC tumors were dosed daily with either vehicle, 30 mg/kg GSK2141795, 0.3 mg/kg GSK1120212 or the combination of GSK2141795 plus GSK1120212 at 30 and 0.3 mg/kg, respectively. GSK2141795 and GSK1120212 treatment alone resulted in 31 and 65% TGI, respectively, compared to the vehicle control mice on day 42 of study (Figure 7A). The combination of GSK2141795 plus GSK1120212 resulted in a TGI of 93% at day 42 of study. Continued dosing of the combination of AKT and MEK inhibitors show enhanced tumor growth delay with the two inhibitors together compared to the either single agent alone (Figure 7A).

Similarly, mice bearing CAPAN-2 tumor xenografts and dosed with 30 mg/kg GSK2141795 resulted in minimal TGI (26%) compared to the vehicle control at day 69, while GSK1120212 resulted in 90% TGI (Figure 7B). The two agents together resulted in an increased TGI of >100%, which is suggestive of tumor regression at day 69 (Figure 7B).

To assess the mechanism of this inhibitor combination, HPAC tumors treated for three days with vehicle, GSK2141795, GSK1120212 or the two agents in combination were harvested, fixed and analyzed by IHC for various markers (Figure 7C). Quantification of proliferation and apoptosis were measured with the Ki67 and cleaved caspase 3 (CC3) assays, respectively. Both AKT and MEK inhibitors given alone or in combination showed a reduction in proliferating cells (Ki67+) following treatment, and up to a 200% increase in apoptosis (CC3 assay). ERK is phosphorylated by MEK and as expected MEK inhibitor treatment resulted in a decrease in phosphorylated ERK in HPAC tumors, whereas AKT inhibitor had no effect. AKT inhibition causes feedback hyperphosphorylation of AKT and this was observed in HPAC xenografts. This effect was similar in mice treated with GSK2141795 alone or in combination with GSK1120212. PRAS40 and ribosomal S6 phosphorylation showed a decrease with both MEK and AKT inhibitor treatment, with additive decrease when the two drugs were given together (Figure 7C).

## Discussion

We describe the biochemical and biological characterization of two orally available, small molecule inhibitors of the AKT kinases that have recently completed Phase I dose escalation studies in cancer patients [18], [19]. GSK2110183 and GSK2141795 have very similar characteristics; both are potent inhibitors of the AKT kinases with sub-nanomolar potency against AKT1 and low nanomolar potency against AKT2 and 3. As with most ATP-competitive kinase inhibitors, these compounds inhibit some additional kinases, most notably PKA and PKG1α (Table 1). Thus, it cannot be ruled out that inhibition of these additional kinases may contribute to some of the cellular phenotypes observed with these molecules. Activating mutations (E17K) in the pleckstrin homology domain of *AKT1* and *AKT3* have been reported in various malignancies [20]–[22]. These activating mutations confer a constitutive activation of the kinase activity. More recently, E17K mutation in *AKT1* has been associated with Proteus syndrome and a similar mutation in *AKT2* was found in patients with rare hypoglycemia [23], [24]. GSK2110183 and GSK2141795 inhibit the activity of AKT1 E17K mutant with IC_50_ comparable to the wild type, which is consistent with their mode of inhibition in the ATP pocket, away from the PH domain. This clearly differentiates these molecules from allosteric AKT inhibitors which bind to the PH domain and are inactive against these AKT mutants [25]. Activity against PH domain mutant AKT provides potential opportunity to evaluate these compounds in cancer patients with these mutations. Once the human safety profile has been well established, it will be tempting to explore them in patients with Proteus syndrome and/or hypoglycemia caused by *AKT1* or *AKT2* mutation, respectively.

Cellular potencies of GSK2110183 and GSK2141795 are comparable, although the latter is 2–3 times more potent in general in most of the experiments and against a large cell panel (Table S2). Consistent with inhibition of phosphorylation of various AKT substrates, a feedback hyperphosphorylation of AKT at both Ser473 and Thr308 was observed as shown before by multiple investigators [12], [15]. Translocation of a GFP tagged FOXO3A to the nucleus is apparent in the presence of both molecules, showing the functional consequence of change in phosphorylation of this transcription factor.

Potent activity against AKT kinases and relative selectivity against a large kinase panel translates in cells as increased sensitivity of tumor cell lines with *PIK3CA* and/or *PTEN* mutations, genes known to regulate AKT pathway activation. Mutation in *KRAS* or *BRAF* results in activation of MAPK pathway, which was not altered by AKT inhibitors (Figure 1A) and as such cell lines harboring these mutations were generally less sensitive to compounds in proliferation assays. Among hematological tumors, cell lines from B and T cell origin were more sensitive compared to myeloid lineage, the latter being associated with MAPK pathway activation [26], [27].

AKT inhibitors demonstrated a dose-dependent decrease in PRAS40 phosphorylation in BT474 tumor xenografts *in vivo* that is sustained for 8–24 h with once daily administration. In general, approximately 2–4 µM of compound in blood (C_max_) achieves ∼60% inhibition of phospho-PRAS40. Both molecules had dose-dependent and proportional PK in mice, with tumor tissue concentrations ≥3-fold higher than blood. Certainly, a higher exposure of compound is required to inhibit Akt activity in vivo than in the various in vitro assays, which is consistent with high protein binding (>95% in human and rodent plasma) for both compounds. High protein binding and/or cellular permeability are likely contributing to the shift from biochemical to cellular activity, as the latter is generally performed with 5–10% serum in culture medium. Anti-tumor efficacy of the molecules was tested in several mouse models of human cancer including BT474 and SKOV3 (ovarian) both of which contain *PIK3CA* mutations (H1047R or K111N) and amplification of *ERBB2* rendering the AKT pathway hyperactive. *In vitro*, both cell lines were sensitive to the molecules with EC_50_'s ranging from 66 nM to 1.1 µM. Maximal inhibition of BT474 tumor growth of 61 and 98% was achieved with 30 mg/kg GSK2110183 and 100 mg/kg GSK2141795 over 3 weeks, respectively. The SKOV3 tumor xenograft also showed robust TGI of 80 and 93% following administration of 30 mg/kg GSK2110183 and 100 mg/kg GSK2141795.

Since AKT is essential to insulin receptor signaling and glucose uptake, the effect of compound administration on blood glucose and insulin levels in mice was evaluated. GSK2141795, the more potent (and also with more off-target kinase inhibition) of the two compounds in cell and *in vivo* studies induces the largest change in blood glucose of between 200–300 mg/dL when dosed at the maximal tolerated dose of 30 mg/kg, daily. As expected, both molecules show an increase in circulating insulin level that mirrors the glucose effects. At low doses, hyperinsulinemia occurs prior to glucose elevation suggesting changes in insulin level could be used clinically to predict the approach to a clinically relevant dose (data not shown). The previously reported AKT inhibitor, GSK690693 induced a much larger increase in hyperglycemia of ∼500 mg/dL in mice [12]. Analysis of the kinase activity of GSK690693 compared to GSK2110183 and GSK2141795 shows a clear divergence of activity on key kinases involved in insulin signaling and glucose homeostasis including AMPK and several PKC isoforms. Lack of activity of these new molecules on such kinases may explain the reduced effect on glucose control.

Based on the potent and selective AKT kinase inhibition and overall pharmaceutical properties, both GSK2110183 and GSK2141795 are being evaluated in patients with various cancers. Although some patients showed partial response and stable disease on treatment with single agent GSK2141795 in the dose escalation study [18], the overall clinical activity was modest in this unselected patient population, consistent with the results from other agents targeting PI3K-AKT pathway [8]. Mutations in *KRAS* or *BRAF* are associated with resistance to AKT inhibitors and inhibition of PI3K/AKT/mTOR signaling may lead to activation of the MEK/ERK signaling [28]–[30] which can confer resistance to PI3K pathway inhibitors [30]–[32]. Similarly, MEK inhibition leads to feedback regulation of AKT signaling and attenuation of sensitivity to MEK inhibitors [32]–[36]. Given the relatively high frequency (∼90%) of *KRAS* mutation in pancreatic cancer, we chose CAPAN-2 and HPAC tumor xenografts harboring distinct mutations in *KRAS*, namely G12D and G12V, respectively for the combination studies. Enhanced anti-tumor efficacy in both models was associated with greater reduction in phospho-S6 level, a measure of mTORC1 inhibition which has recently been shown to be a better predictor of sensitivity to both PI3K and MAPK pathway inhibitors in preclinical models as well as in patients with different tumors [37]–[38]. Combination of small molecule PI3K/AKT and MEK inhibitors have been challenging due to increased toxicity in patients and will likely require identifying the optimal dose and schedule as well as careful selection of patients likely to benefit from these combinations [8]. Based on the promising preclinical activity, a clinical study combining GSK2141795 with trametinib (GSK1120212) is ongoing to evaluate various dosing schedules with pharmacodynamic marker assessment for both PI3K/AKT and MAPK signaling, including phospho-S6 measurement in paired biopsies. Modulation of pharmacodynamic markers will be key to identifying a clinically meaningful therapeutic dose and schedule for the combination.

## Supporting Information

Figure S1 **Chemical structures of GSK2110183 and GSK2141795.**
(TIF)

Figure S2 **Effect of GSK2141795 and GSK2110183 on the nuclear translocation of FOXO3A.** *A*, MDA-MB-468 cells, stably expressing a FOXO3A-GFP reporter gene, were treated with vehicle, GSK2110183 (1 µM) or GSK2141795 (1 µM). *B*, Titration of GSK2110183 and GSK2141795 at various concentration for 1 h and cells analysed on a high content imager to generate inhibition curves and EC50's. *C*, Western analysis for phosphorylated Foxo3a was performed on protein lysate 1 h after compound treatment and tublin was used as a loading control.
(TIF)

Figure S3 **Time course PD/PK relationship of GSK2110183 and GSK2141795 in BT474 tumor xenografts.** Female SCID mice bearing BT474 tumors were treated with vehicle, 100 mg/kg GSK2110183 (A) or 30 mg/kg GSK2141795 (B) daily for 7 days (QDx7; n = 3/group). Tumors and blood were harvested over time at 0, 1, 2, 4, 6, 8, 24, 36, 48 and 72 h post the last dose. Tumors were analysed by ELISA for phosphorylated and total PRAS40 levels. The concentration of drug in the tumor (black triangles; ng/g) and blood (black squares; ng/mL) was quantified by LC/MS-MS. Data represents mean ± s.d. #p<0.05, *p<0.01.
(TIF)

Figure S4 **Anti-tumor activity of GSK2110183 in vivo.** Female mice bearing either BT474 (*A*) or SKOV3 (*B*) tumors were treated with vehicle (black line) or GSK2110183 at 10 (red line), 30 (blue line) or 100 (gold line) mg/kg once daily for 21 days (QDx21). Duration of treatment is shown by the horizontal grey line. Tumor volume was measured twice per week. Data represents mean ± SEM. #p<0.05, *p<0.01.
(TIF)

Table S1 **Kinase selectivity of GSK2110183 and GSK2141795.**
(XLSX)

Table S2 **Anti-proliferative activity of GSK2110183 and GSK2141795 against solid tumor cell lines.**
(XLSX)

Table S3 **Anti-proliferative activity of GSK2110183 and GSK2141795 against hematological tumor cell lines.**
(XLSX)

Methods S1 **Supplemental methods.**
(DOCX)

## References

[1] Yuan TL, Cantly LC (2008) PI3K pathway alterations in cancer: variations on a theme. Oncogene 27: 5497–510. 18794884 10.1038/onc.2008.245 PMC3398461

[2] Alessi DR, James SR, Downes CP, Holmes AB, Gaffney PR, et al (1997) Characterization of a 3-phosoinositude-dependant protein kinase which phosphorylates and activates protein kinase B alpha. Curr Biol 7: 261–9. 9094314 10.1016/s0960-9822(06)00122-9

[3] Currie RA, Walker KS, Gray A, Deak M, Casamayor A, et al (1999) Role of phosphatidylinositol 3, 4, 5- triphosphate in regulating the activity and localization of 3-phosphoinositide-dependant protein kinase-1. Biochem J 337: 575–83. 9895304 PMC1220012

[4] Majumder PK, Sellers WR (2005) Akt regulated pathways in prostate cancer. Oncogene 24: 7465–74. 16288293 10.1038/sj.onc.1209096

[5] Hresko RC, Mueckler M (2005) mTOR/Rictor is the Ser 473 kinase for Akt/protein kinase B in 3T3-L1 adipocytes. J Biol Chem 280: 40406–16. 16221682 10.1074/jbc.M508361200

[6] Sarbassov DD, Ali SM, Sengupta S, Sheen JH, Hsu PP, et al (2006) Prolonged Rapamycin treatment inhibits mTORC2 assembly and Akt/PKB. Mol Cell 22: 159–68. 16603397 10.1016/j.molcel.2006.03.029

[7] Sarbassov DD, Guertin DA, Ali SM, Sabatini DM (2005) Phosphorylation and regulation of Akt/PKB by the Rictor-mTOR complex. Science 307: 1098–101. 15718470 10.1126/science.1106148

[8] Rodon J, Dienstmann R, Serra V, Tabernero J (2013) Development of PI3K inhibitors: lessons learned from early clinical trials. Nat Rev Clin Oncol 10: 143–153. 23400000 10.1038/nrclinonc.2013.10

[9] Lin J, Sampath D, Nannini MA, Lee BB, Degtyarev M, et al (2012) Targeting activated Akt with GDC-0068, a novel selective Akt inhibitor that is efficacious in multiple tumor models. Clin Cancer Res 19: 1760–72. 10.1158/1078-0432.CCR-12-3072 23287563

[10] Davies BR, Greenwood H, Dudley P, Crafter C, Yu DH, et al (2012) Preclinical pharmacology of AZD5363, an inhibitor of AKT: pharmacodynamics, antitumor activity, and correlation of monotherapy activity with genetic background. Mol Cancer Ther 11: 873–87. 22294718 10.1158/1535-7163.MCT-11-0824-T

[11] Saleh M, Papadopoulos K, Arabnia A, Patnaik A, Stein RM, et al. (2013) First in human study with ARQ 092, a novel pan AKT-inhibitor: Results from the advanced solid tumor cohorts. Am Assoc Cancer Res Annual Meet LB-197.

[12] Rhodes N, Heerding DA, Duckett DR, Eberwein DJ, Knick VB, et al (2008) Characterization of an AKT kinase inhibitor with potent pharmacodynamic and antitumor activity. Cancer Res 68: 2366–74. 18381444 10.1158/0008-5472.CAN-07-5783

[13] Yap TA, Yan L, Patnaik A, Fearen I, Olmos D, et al (2011) First-in-human clinical trial of the oral pan-AKT inhibitor MK-2206 in patients with advanced solid tumors. J Clin Oncol 29: 4688–95. 22025163 10.1200/JCO.2011.35.5263

[14] Gilmartin AG, Bleam MR, Groy A, Moss KG, Minthorn EA, et al (2011) GSK1120212 (JTP-74057) is an inhibitor of MEK activity and activation with favorable pharmacokinetic properties for sustained in vivo pathway inhibition. Clin Cancer Res 17: 989–1000. 21245089 10.1158/1078-0432.CCR-10-2200

[15] Han EK-H, Leverson JD, McGonigal T, Shah OJ, Wood KW, et al (2007) Akt inhibitor A-443653 induced rapid Akt Ser-473 phosphorylation independent of mTORC1 inhibition. Oncogene 26: 5655–61. 17334390 10.1038/sj.onc.1210343

[16] Takaishi H, Konishi H, Matsuzaki H, Ono Y, Shirai Y, et al (1999) Regulation of nuclear translocation of Forkhead transcription factor AFX by protein kinase B. PNAS 96: 11836–41. 10518537 10.1073/pnas.96.21.11836 PMC18373

[17] Sos M, Fischer S, Ullrich R, Peifer M, Heuckmann JM, et al (2009) Identifying genotype-dependent efficacy of single and combined PI3K- and MAPK- pathway inhibition in cancer. PNAS 106: 18351–56. 19805051 10.1073/pnas.0907325106 PMC2757399

[18] Burris HA, Siu LL, Infante JR, Wheler JJ, Kurkjian C, et al (2011) Safety, pharmacokinetics (PK), pharmacodynamics (PD), and clinical activity of the oral AKT inhibitor GSK2141795 (GSK795) in a phase I first-in human study. J Clin Oncol 29 suppl: 2003.

[19] Spencer A, Yoon S-S, Harrison SJ, Morris S, Smith D, et al. (2011) Novel AKT inhibitor GSK2110183 shows favorable safety, pharmacokinetics, and clinical activity in Multiple Myeloma. Preliminary results from a phase I first-time-in-human study. Proc 53^rd^ Am Soc Hematol Annual Meet; Dec10-13; San Diego, CA Abstract 1856.

[20] Carpten JD, Faber AL, Horn C, Donoho GP, Briggs SL, et al (2007) A transforming mutation in the pleckstrin homology domain of Akt 1 in cancer. Nature 448: 439–44. 17611497 10.1038/nature05933

[21] Davies MA, Sternke-Hale K, Tellez C, Calderone TL, Deng W, et al (2008) A novel Akt 3 mutation in melanoma tumors and cell lines. Br J Cancer 99: 1265–8. 18813315 10.1038/sj.bjc.6604637 PMC2570525

[22] Shoji K, Oda K, Nakagawa S, Hosokawa S, Nagae G, et al (2009) The oncogenic mutation in plekstrin homology domain of Akt 1 in endometrial carcinomas. Br J Cancer 101: 145–8. 19491896 10.1038/sj.bjc.6605109 PMC2713716

[23] Lindhurst MJ, Sapp JC, Teer JK, Johnston JJ, Finn EM, et al (2011) A mosaic activating mutation in AKT1 associated with the Proteus Syndrome. New Eng J Med 365: 611–9. 21793738 10.1056/NEJMoa1104017 PMC3170413

[24] Hussain K, Challis B, Rocha N, Payne F, Minic M, et al (2011) An activating mutation of AKT2 and human hypoglycemia. Science 334: 474. 21979934 10.1126/science.1210878 PMC3204221

[25] Wu W-I, Voegtli WC, Sturgis HL, Dizon FP, Vigers GPA, et al (2010) Crystal structure of human AKT1 with an allosteric inhibitor reveals a new mode of kinase inhibition. PLoS One 9: e12913. 10.1371/journal.pone.0012913 PMC2944833 20886116

[26] Morgan MA, Dolp O, Reuter CWM (2001) Cell-cycle-dependant activation of mitogen-activated protein kinase kinase (MEK1/2) in myeloid leukemia cell lines and induction of growth inhibition and apoptosis by inhibitors of RAS signaling. Neoplasia 97: 1823–34. 10.1182/blood.v97.6.1823 11238126

[27] Ricciardi MR, Scerpa MC, Bergamo P, Ciuffreda L, Petrucci MT, et al (2012) Therapeutic potential of MEK inhibition in acute myelogenous leukemia: rationale for “vertical” and “lateral” combination studies. J Mol Med 90: 1133–44. 22399013 10.1007/s00109-012-0886-z

[28] Ebi H, Costa C, Faber AC, Nishtala M, Kotani H, et al (2013) PI3K regulates MEK/ERK signaling in breast cancer via the Rac-GEF, P-Rex1. PNAS 52: 21124–9. 10.1073/pnas.1314124110 PMC3876254 24327733

[29] Carracedo A, Ma L, Teruya-Feldstein J, Rojo F, Salmena L, et al (2008) Inhibition of mTORC1 leads to MAPK pathway activation through a PI3K-dependent feedback loop in human cancer. J Clin Invest 118: 3065–3074. 18725988 10.1172/JCI34739 PMC2518073

[30] Serra V, Scaltriti M, Prudkin L, Eichhorn PJA, Ibrahim H, et al (2011) PI3K inhibition results in enhanced HER signaling and acquired ERK dependency in HER2-overexpressing breast cancer. Oncogene 30: 2547–2557. 21278786 10.1038/onc.2010.626 PMC3107390

[31] Chandarlapaty S, Sawai A, Scaltriti M, Rodrik-Outmezguine V, Grbovic-Huezo O, et al (2011) AKT inhibition relieves feedback suppression of receptor tyrosine kinase expression and activity. Cancer Cell 19: 58–71. 21215704 10.1016/j.ccr.2010.10.031 PMC3025058

[32] Faber AC, Li D, Song Y, Liang MC, Yeap BY, et al (2009) Differential induction of apoptosis in HER2 and EGFR addicted cancers following PI3K inhibition. Proc Natl Acad Sci U S A 106: 19503–8. 19850869 10.1073/pnas.0905056106 PMC2765921

[33] Turke AB, Song Y, Costa C, Cook R, Arteaga CL, et al (2012) MEK inhibition leads to PI3K/AKT activation be relieving a negative feedback on ERBB receptors. Cancer Res 72: 1–10. 10.1158/0008-5472.CAN-11-3747 PMC3515079 22552284

[34] Hoeflich KP, O'Brien C, Boyd Z, Cavet G, Guerrero S, et al (2009) In vivo antitumor activity of MEK and phosphatidylinositol 3-kinase inhibitors in basal-like breast cancer models. Clin Cancer Res 15: 4649–64. 19567590 10.1158/1078-0432.CCR-09-0317

[35] Mirzoeva OK, Das D, Heiser LM, Bhattacharya S, Siwak D, et al (2009) Basal subtype andMAPK/ERK kinase (MEK)-phosphoinositide 3-kinase feedback signaling determine susceptibility of breast cancer cells to MEK inhibition. Cancer Res 69: 565–72. 19147570 10.1158/0008-5472.CAN-08-3389 PMC2737189

[36] Yoon YK, Kim HP, Han SW, Hur HS, Oh do Y, et al (2009) Combination of EGFR and MEK1/2 inhibitor shows synergistic effects by suppressing EGFR/HER3-dependent AKT activation in human gastric cancer cells. Mol Cancer Ther 8: 2526–36. 19755509 10.1158/1535-7163.MCT-09-0300

[37] Elkabets M, Vora S, Juric D, Morse N, Mino-Kenudson M, et al. (2013) mTORC1 inhibition is required for sensitivity to PI3K p110α inhibitors in *PIK3CA*-mutant breast cancer. Sci. Transl. Med. 5, 196ra99. 10.1126/scitranslmed.3005747 PMC3935768 23903756

[38] Corcoran RB, Rothenberg SM, Hata AN, Faber AC, Piris A, et al. (2013) TORC1 suppression predicts responsiveness to RAF and MEK inhibition in *BRAF*-mutant melanoma. Sci. Transl. Med. 5, 196ra98. 10.1126/scitranslmed.3005753 PMC3867020 23903755

