# Supplementary figures and images for: Discovery of Novel AKT Inhibitors with Enhanced Anti-Tumor Effects in Combination with the MEK Inhibitor
Source: PLoS One. 2014 Jun 30;9(6):e100880. doi: 10.1371/journal.pone.0100880 (PMC4076210; doi:10.1371/journal.pone.0100880)

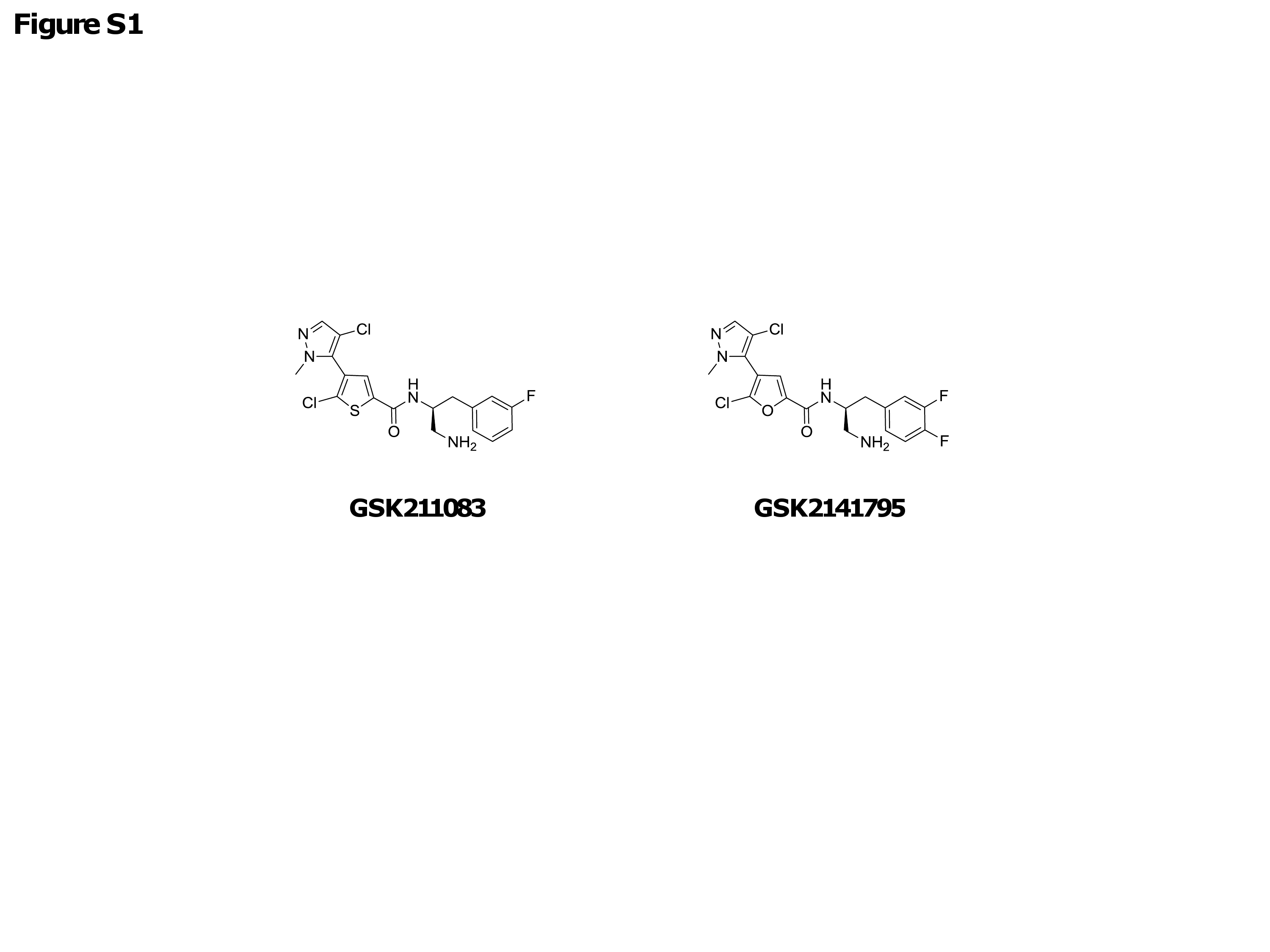

Supplement: Figure S1 — Chemical structures of GSK2110183 and GSK2141795. (TIF) [file pone.0100880.s001.tif]

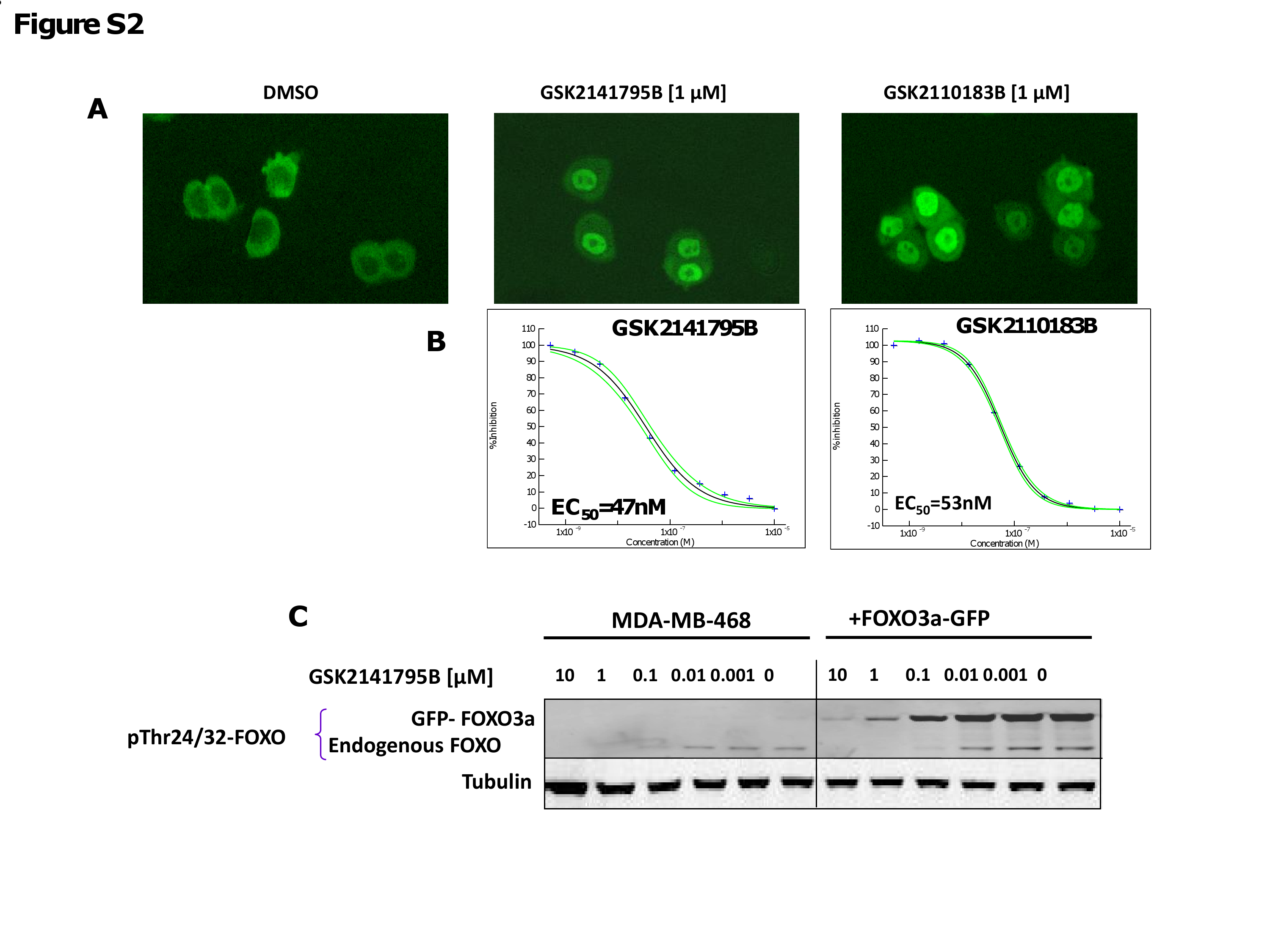

Supplement: Figure S2 — Effect of GSK2141795 and GSK2110183 on the nuclear translocation of FOXO3A. A, MDA-MB-468 cells, stably expressing a FOXO3A-GFP reporter gene, were treated with vehicle, GSK2110183 (1 µM) or GSK2141795 (1 µM). B, Titration of GSK2110183 and GSK2141795 at various concentration for 1 h and cells analysed on a high content imager to generate inhibition curves and EC50's. C, Western analysis for phosphorylated Foxo3a was performed on protein lysate 1 h after compound treatment and tublin was used as a loading control. (TIF) [file pone.0100880.s002.tif]

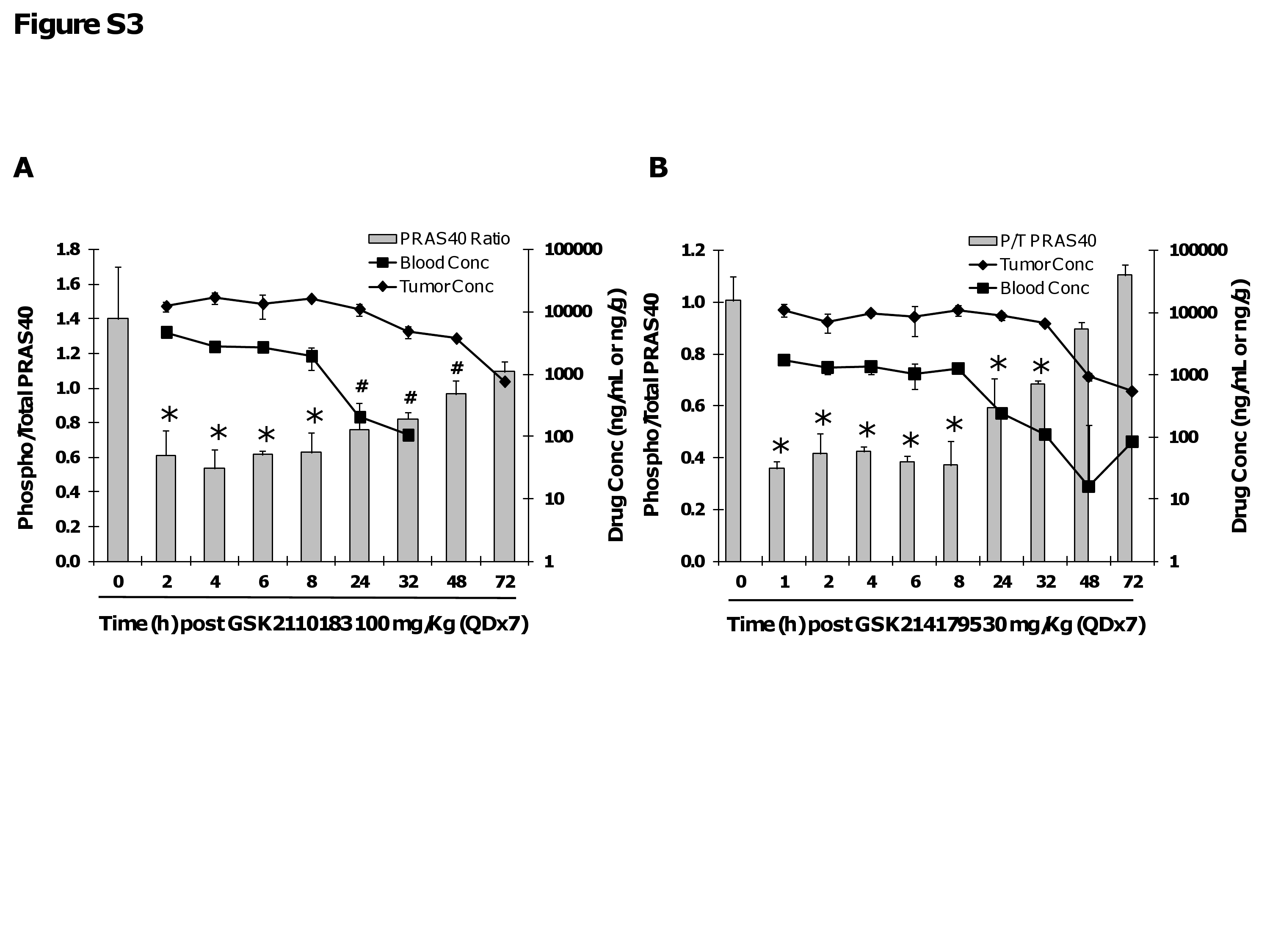

Supplement: Figure S3 — Time course PD/PK relationship of GSK2110183 and GSK2141795 in BT474 tumor xenografts. Female SCID mice bearing BT474 tumors were treated with vehicle, 100 mg/kg GSK2110183 (A) or 30 mg/kg GSK2141795 (B) daily for 7 days (QDx7; n = 3/group). Tumors and blood were harvested over time at 0, 1, 2, 4, 6, 8, 24, 36, 48 and 72 h post the last dose. Tumors were analysed by ELISA for phosphorylated and total PRAS40 levels. The concentration of drug in the tumor (black triangles; ng/g) and blood (black squares; ng/mL) was quantified by LC/MS-MS. Data represents mean ± s.d. #p<0.05, *p<0.01. (TIF) [file pone.0100880.s003.tif]

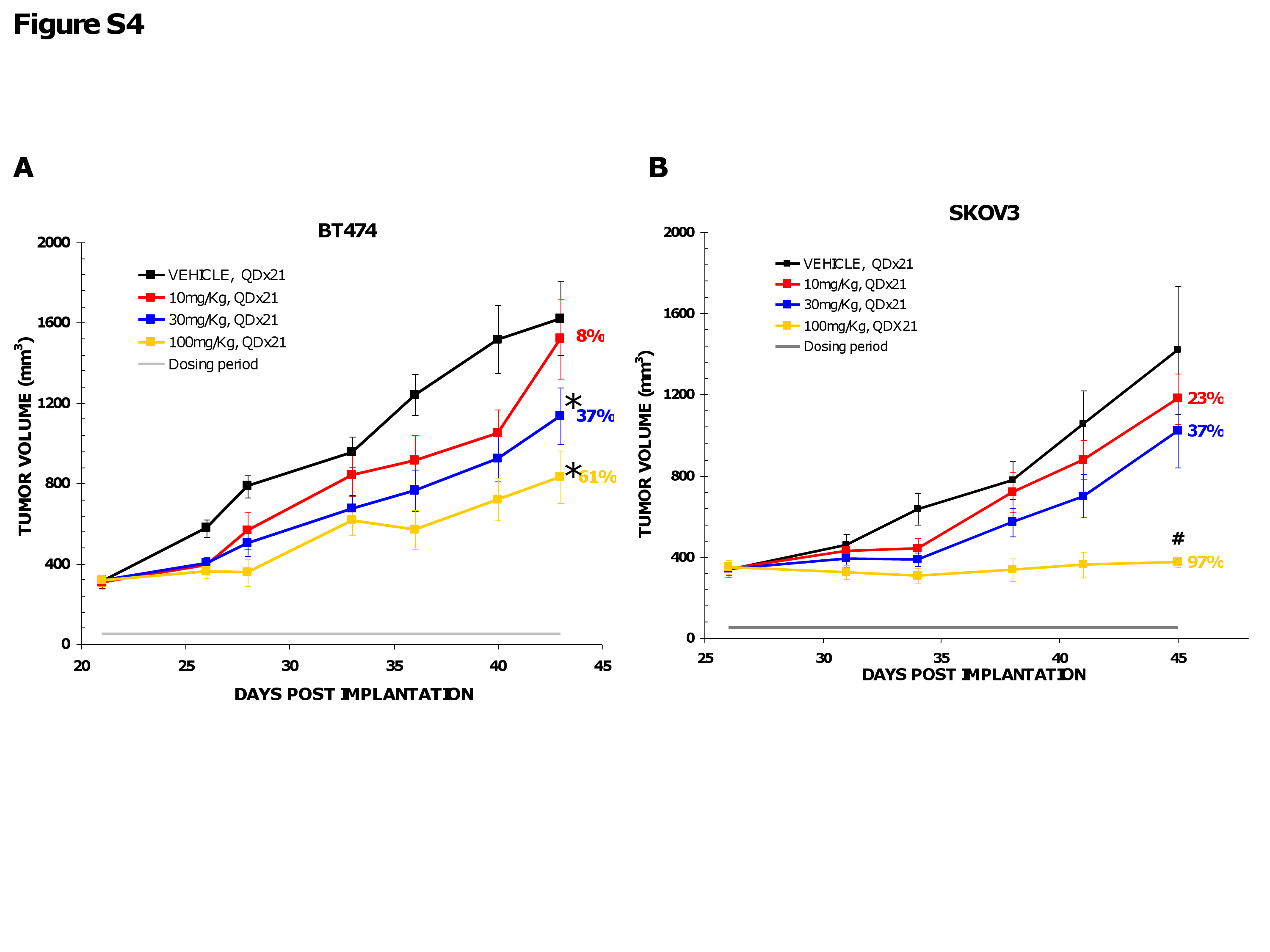

Supplement: Figure S4 — Anti-tumor activity of GSK2110183 in vivo. Female mice bearing either BT474 (A) or SKOV3 (B) tumors were treated with vehicle (black line) or GSK2110183 at 10 (red line), 30 (blue line) or 100 (gold line) mg/kg once daily for 21 days (QDx21). Duration of treatment is shown by the horizontal grey line. Tumor volume was measured twice per week. Data represents mean ± SEM. #p<0.05, *p<0.01. (TIF) [file pone.0100880.s004.tif]
