# Supplementary material for: Discovery of Novel AKT Inhibitors with Enhanced Anti-Tumor Effects in Combination with the MEK Inhibitor
Source: PLoS One. 2014 Jun 30;9(6):e100880. doi: 10.1371/journal.pone.0100880 (PMC4076210; doi:10.1371/journal.pone.0100880)
Supplement: Methods S1 — Supplemental methods. (DOCX) [file pone.0100880.s008.docx]

**Discovery of novel AKT inhibitors with enhanced anti-tumor effects in combination with the MEK inhibitor**

Melissa Dumble^1^, Ming-Chih Crouthamel^1^, Shu-Yun Zhang^1^, Michael Schaber^2^, Dana Levy^1^, Kimberly Robell^1^, Qi Liu^1^, David J. Figueroa^1^, Elisabeth A. Minthorn^1^, Mark A. Seefeld^1^, Meagan B. Rouse^1^, Sridhar K. Rabindran^1^, Dirk A. Heerding^1^, Rakesh Kumar^1^

GlaxoSmithKline, ^1^Oncology R&D, Collegeville, PA; ^2^Platform Technology & Science, Collegeville, PA

**Supplemental Methods:**

**Translocation Assay**

MDA-MB-468 cells stably transfected with FOXO3A-GFP were plated at 1000 cells/96 well and allowed to adhere overnight. Cells were treated with DMSO or AKT inhibitor (0-10 μM) for 1 h. Plates were centrifuged at 1200 rpm for 10 min, and the medium aspirated. Cells were fixed with 200 μL of 4% paraformaldehyde (Alfa Aesar, Wood Hill, MA), and DAPI (80 ng/mL, Roche, Branchburg, NJ) was added to each well and incubated for 20 min at room temperature. To determine EC_50_ values, cells were washed, imaged (10x objective) and analyzed using the InCell Analyzer 1000 (DAPI 360/460 excitation filter, GFP 480/535 emission filters, 51008bs dichroic mirror; (GE Piscataway, NJ)). Five fields per well were imaged to achieve ≥250 nuclei count per well. A ratio of nuclear to cytoplasmic intensity was calculated by the software. The formula used to calculate percentage nuclear translocation = (Fluorescence _sample_- Fluorescence _DMSO_)/ (Fluorescence _10 μM treatment sample_- Fluorescence _DMSO_)*100. The calculation produces a scale of 0-100% with 100% being 10 μM of compound treatment and 0 being DMSO treatment. The normalized values were then plotted in the XLFit 4 parameter curve fitting program to generate EC_50_ values.

**Cell Cycle Analysis**

Cells were plated in 96 well plates at a density of 3000-5000 cells/well and allowed to adhere overnight. Cells were treated with DMSO or various concentrations of drug for 72 h, fixed and stained with propidium iodide to stain nuclei (Vindelov, 1982). Cells were analyzed in a flow cytometer (FACSCalibur; BD Biosciences, Franklin Lakes, NJ) and the fraction of cells in each phase of cell cycle was determined using the cell cycle analysis platform contained within the FlowJo software (Tree Star, Ashland, OR).

***In vivo* time course pharmacodynamic assay**

The pharmacodynamic studies were carried out in SCID mice bearing BT474 tumor xenografts. Mice were dosed with either vehicle, GSK2110183 or GSK2141795 daily for 7 days prior to harvesting tumor and blood at several time points out to three days (n=3/ group). Protein lysates were prepared and the phospho-PRAS40 ELISA was performed on tumor samples according to the methods described in the main text. Concentration of the test compounds was analysed by HPLC/MS/MS analysis.
